# Scleral Buckle Extrusion Associated with Phthisis

**DOI:** 10.1155/2011/942946

**Published:** 2011-12-20

**Authors:** Tural Galbinur, Itay Chowers

**Affiliations:** Department of Ophthalmology, Hebrew University-Hadassah Schools of Medicine and Dental Medicine, P.O. Box 12000, Jerusalem 91120, Israel

## Abstract

Exposure of implanted episcleral element is a rare complication of buckling procedures. We describe a 40-year-old man who presented to our clinic complaining of foreign body sensation and irritation in his left eye which lasted several months. The patient history was positive for bilateral rhegmatogenous retinal detachment which was treated with sclera buckling. Upon presentation the left eye demonstrated phthisis and an exposed and infected sclera buckle and explant in the lower quadrants. The explant was removed, and the patient was treated with antibiotics. This case suggests that wide encircling sclera element might erode through the conjunctiva of eyes undergoing phthisis. Integrity of the conjunctiva overlying episcleral implant should be evaluated during routine follow-up exams to exclude exposure of the implant particularly in eyes undergoing phthisis.

## 1. Introduction

Extrusion and infection of the silicone rubber implant is a rare complication of scleral buckling procedures [1–3]. We report a case of large extrusion of such implant through the conjunctiva in association with phthisis bulbi.

## 2. Case Report

A 40-year-old man presented with foreign body sensation, hyperemic swollen conjunctiva, and discharge in his left eye which lasted for several months prior to his presentation. Ocular history was significant for bilateral rhegmatogenous retinal detachment three years prior to his presentation which were treated at another institution. At that time the right eye underwent successful scleral buckling procedure. The left eye underwent scleral buckling procedure with placement of 360° encircling explant followed by vitrectomy. Following that procedure retinal attachment was not obtained and phthisis of the left eye developed.

**Figure 1:**
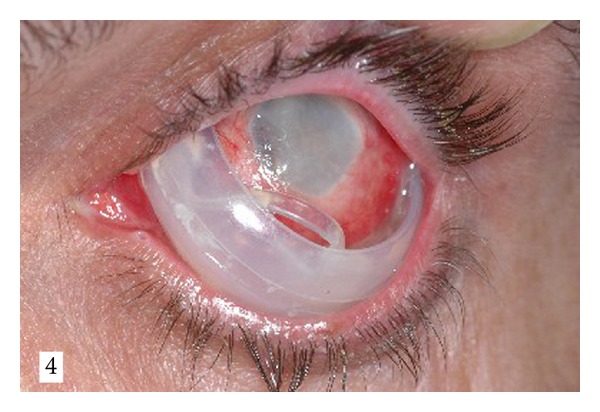
Photograph of the left eye at the time of presentation. Exposure of silicon band and tire implant is seen thorough the inferior quadrants.

Upon examination on presentation, visual acuity was 20/125 in the right eye and no light perception in the left eye. Anterior segment was unremarkable in the right eye, and ophthalmoscopy revealed attached retina with indentation of scleral buckle 360°. The left eye demonstrated phthisis with hyperemic and chemotic conjunctiva and extensive yellowish discharge. An exposed band and tire were seen eroding through the conjunctiva (Figure 1). Conjunctival fistula was present throughout the inferior meridians. A 360° band and 360° tire were removed, and the patient was treated with topical and systemic antibiotics. Skin flora including both *Gram-positive and Gram-negative *bacteria was isolated from the implant.

## 3. Discussion

Among the infrequent complications associated with scleral buckle implants are inflammation, infection, extrusion, and intrusion [1–4]. Nguyen and colleagues [3] identified only four patients from their practices during a 20-year period that had erosion or intrusion of silicone rubber scleral buckle implants out of approximately 4400 cases.

The case presented here suggests a potential association between exposure of wide scleral implants and phthisis. Presumably, wide encircling implants from the type which was used in this case are relatively inflexible and resist the force induced by the shrinking phthitic eye. While the radius of a phthitic eye gradually decreases, such implants might retain their larger radius and erode through the conjunctiva.

This case suggests that integrity of the conjunctiva overlying episcleral implant in general and wide tire in particular should be evaluated during routine follow-up exams to exclude exposure of the implant particularly in eyes undergoing phthisis.
